# Predicting Structure-Function Relations and Survival following Surgical and Bronchoscopic Lung Volume Reduction Treatment of Emphysema

**DOI:** 10.1371/journal.pcbi.1005282

**Published:** 2017-02-09

**Authors:** Jarred R. Mondoñedo, Béla Suki

**Affiliations:** 1 Department of Biomedical Engineering, Boston University, Boston, MA, United States of America; 2 School of Medicine, Boston University, Boston, MA, United States of America; University of Oxford, UNITED KINGDOM

## Abstract

Lung volume reduction surgery (LVRS) and bronchoscopic lung volume reduction (bLVR) are palliative treatments aimed at reducing hyperinflation in advanced emphysema. Previous work has evaluated functional improvements and survival advantage for these techniques, although their effects on the micromechanical environment in the lung have yet to be determined. Here, we introduce a computational model to simulate a force-based destruction of elastic networks representing emphysema progression, which we use to track the response to lung volume reduction via LVRS and bLVR. We find that (1) LVRS efficacy can be predicted based on pre-surgical network structure; (2) macroscopic functional improvements following bLVR are related to microscopic changes in mechanical force heterogeneity; and (3) both techniques improve aspects of survival and quality of life influenced by lung compliance, albeit while accelerating disease progression. Our model predictions yield unique insights into the microscopic origins underlying emphysema progression before and after lung volume reduction.

## Author Summary

Surgical and, more recently, bronchoscopic lung volume reduction is the only available treatments for patients with advanced stage emphysema. Several large-scale, clinical studies have outlined appropriate selection criteria based on patient outcomes; however, the underlying mechanisms determining disease progression and response to these treatments are not well-understood. To answer this question, we have developed a network model of the lung to compare immediate and long-term response to each treatment. This approach allows us to directly study macroscopic changes in function related to microscopic changes in the local structural and mechanical environment. In addition, it facilitates direct comparisons between surgical and bronchoscopic lung volume reduction given identical initial conditions, which is not feasible in a clinical study. We propose here a mechanism suggesting that lung volume reduction efficacy is intimately linked to changes in microscopic force heterogeneity within the lung. Such an understanding of the mechanisms driving emphysema has the potential to greatly improve current therapies for this condition through more rationalized, patient-specific treatment strategies.

## Introduction

Emphysema, a subtype of chronic obstructive pulmonary disease (COPD), is a progressively destructive lung tissue disease characterized by abnormal and permanent enlargement of airspaces distal to the terminal bronchioles. This largely preventable, yet presently incurable disease is associated with high morbidity and mortality, presenting a substantial burden on resource utilization [1,2]. While pharmacological treatment can have limited benefits for patients, especially in advanced stages of disease, surgical and bronchoscopic treatments aimed at reducing hyperinflated lung volumes have been shown to ameliorate symptoms of dyspnea, improve quality of life, and in certain cases provide a survival advantage [3–6].

Proposed mechanisms by which lung volume reduction results in functional improvements primarily involve a reduction in hyperinflation leading to restored chest wall and diaphragm mechanics, and an increase in elastic lung recoil (hence a decrease in lung compliance) and radial traction on airways leading to improved expiratory flow rates and lung emptying [7,8]. In lung volume reduction surgery (LVRS), emphysematous tissue is removed from the upper lung by wedge excision typically via median sternotomy or video-assisted thoracoscopic surgery. Although LVRS has proven efficacy in patients with predominantly upper-lobe emphysema and low baseline exercise capacity [9], many still do not meet the strict indications for this procedure. Moreover, widespread implementation of LVRS is hindered by high costs, a limited number of highly experienced centers, and significant post-procedural morbidity and mortality [8].

Recently, a growing number of non-surgical techniques have been developed with the goal of providing less invasive alternatives [10]. Among the more extensively investigated of these bronchoscopic lung volume reduction (bLVR) treatments are (1) nitinol coils, which return to a predetermined shape after placement in target airways while retracting the surrounding diseased lung parenchyma [5,11,12]; (2) one-way valves, which facilitate lobar collapse by blocking regional ventilation but permitting emptying of affected areas [6,13,14]; and (3) biomaterial-based lung sealants, which function to block small airways and prevent collateral ventilation pathways inducing absorption atelectasis in the delivered region [15–17]. While recent evidence appears to be trending in support of bLVR and ongoing studies are expected to further define optimal implementations for such approaches [8], the immediate and long-term effects in the lung have yet to be fully understood.

Previous work has focused primarily on the benefits in clinical function and survival outcomes for these treatments and thus, provides no insight into the micromechanical origins leading to such improvements. The aim of this study was to investigate the structure-function relationships before and after lung volume reduction using a computational elastic network model [18–22]. Here, we simulate emphysema progression by removal of elastic elements in the network, and then track changes in network compliance and structure following LVRS and bLVR interventions. Our simulations shed light on the factors contributing to immediate and long-term treatment efficacy as well as predicted outcomes spanning several years. We show that macroscopic improvements in compliance following lung volume reduction are correlated with the microscopic distribution of mechanical forces in the local lung environment. Furthermore, our model predicts similar survival and quality of life benefits for both interventions, indicating how bLVR may be implemented as an effective, less invasive treatment for advanced emphysema.

## Results

### Network Modeling of Emphysema Progression and Lung Volume Reduction

**Fig 1:**
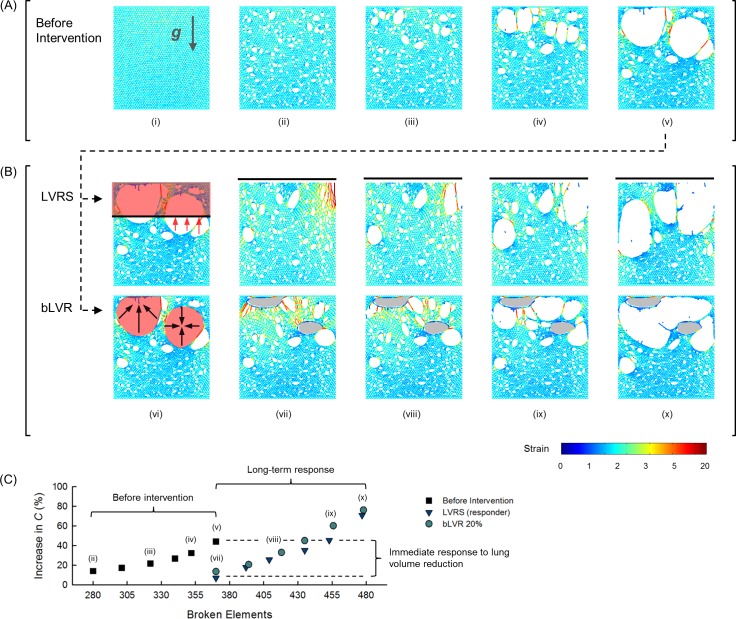
Network Model of Lung Volume Reduction. (A) Representative simulation of emphysema progression before intervention, and (B) comparison of lung volume reduction techniques in a responder network. (i) Initial network configuration representing healthy tissue, direction of gravity indicated by arrow. (ii) Disease progression initiated by randomly breaking ~4% of elements. (iii-v) Gradual network destruction representing tissue failure during emphysema progression. (vi) Illustrations of lung volume reduction techniques initiated from the same disease condition. For LVRS, the upper 30% of the network (red shaded region) was removed and intersected regions were stretched (red arrows) to form a continuous upper border (solid line). For bLVR, affected regions (red shaded areas) were reduced (black arrows) to 20% of their original sizes. (vii-x) Disease progression following either LVRS or bLVR, with reduced regions shown (grey shaded areas). Color bar represents strain distribution of individual elements. (C) Changes in compliance, *C* for the representative network shown.

The elastic behavior of lung tissue was modeled using a two-dimensional (2D) computational network of linearly elastic elements arranged in a hexagonal lattice under the influence of gravity. Disease progression was driven by elimination of network elements carrying a high force, representing *in vivo* tissue failure in regions of high local mechanical stress. Fig 1A shows a representative simulation beginning with healthy tissue followed by gradual deterioration. As tissue failure progressed (*i*.*e*., high force elements were removed), smaller airspaces were observed to coalesce into larger neighboring airspaces representing the process of airspace enlargement characteristic of emphysema progression. Fig 1B illustrates the two lung volume reduction techniques initiated in parallel from the same network configuration. LVRS was simulated by removing the upper portion of the network, while bLVR was simulated by reducing specific affected regions of the network.

To characterize functional changes within the network at each configuration, the network compliance, *C* was calculated as the inverse of the 2D bulk modulus. To characterize structural changes, network heterogeneity was quantified as the coefficient of variation of individual airspace sizes, CV_area_. Prior to lung volume reduction, values of *C* and CV_area_ increased from baseline consistent with the loss of elastic recoil and enlarged airspaces observed in emphysema (Fig 1C). In the representative network shown, both the non-specific LVRS and the region-specific bLVR yielded comparable immediate and long-term improvements in network elasticity as characterized by similar reductions and recoveries in *C*, respectively. In contrast, upper network resection with LVRS was less effective in other networks as characterized by differences in reduction and recovery in *C* (S1 Fig). Thus, variability in initial conditions among the *N* = 14 large networks, representing inter-subject variability, simulated disease distributions with treatment-specific responses to lung volume reduction.

### Immediate Response to Lung Volume Reduction

**Fig 2:**
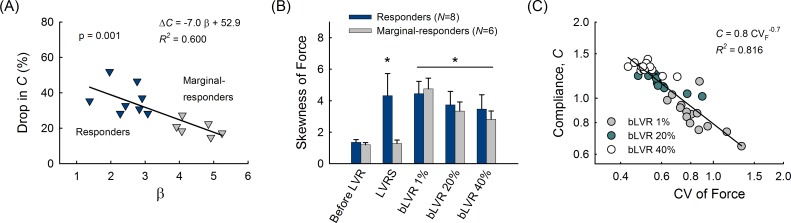
Immediate Response to Lung Volume Reduction. (A) Networks with lower values of β (LVRS responders, dark blue) demonstrated larger changes in compliance, *C* compared to networks with higher values of β (LVRS marginal-responders, grey). (B) Skewness of force distributions before and after lung volume reduction. Error bars represent standard error and *indicates statistically larger compared to before intervention as well as LVRS marginal-responders. (C) Relationship between *C* and the heterogeneity in force distribution, CV_force_ after bLVR reduction to 1% (dark grey), 20% (green), and 40% (open circles) of their original size. A power law, equivalent to a straight line on a log-log graph, fitted the data well with exponent of -0.7 (*R*^*2*^ = 0.816).

To distinguish networks demonstrating a benefit from LVRS, we defined the predictive index β characterizing network heterogeneity below the line of LVRS resection. Here, β was calculated as the coefficient of variation of airspace sizes below the line of LVRS resection. Less heterogeneity in this lower region (*i*.*e*., smaller β) corresponded to relatively spared tissue due to predominately upper lung emphysema. More heterogeneity in this region (*i*.*e*., larger β) corresponded to emphysematous tissue destruction extending into the lower lung regions. Based on this index, an arbitrary threshold of β = 3.5 was defined to divide the networks into LVRS responder (*N* = 8) and marginal-responder (*N* = 6) groups. As shown in Fig 2A, the immediate drop in *C* following LVRS was inversely related to β (*R*^*2*^ = 0.600), with larger changes observed for responders compared to marginal-responders (36.4±8.6% vs. 20.1±4.4%; *p* = 0.001). Representative networks for both groups are shown in the supplement (S2 Fig). In addition, β was inversely related to the overall network heterogeneity quantified by CV_area_ (S2 Fig). That is to say, smaller values of β implied heterogeneous network structure amenable to LVRS, whereas larger values of β implied homogeneous disease patterns less effectively treated by resection of the upper network.

To better characterize the mechanical differences between networks with different β values, we compared the distribution of forces carried by individual network elements before and after intervention. As shown in Fig 2B, LVRS skewed the distribution to the right (*i*.*e*., toward larger forces) for responders, whereas it did not significantly alter the skewness relative to before intervention for marginal-responders. Interestingly, bLVR skewed the distribution of forces to the right for all networks and reduction conditions. As shown in Fig 2C, plotting values of *C* after bLVR against the coefficient of variation of forces (CV_force_) revealed that a simple power law function fitted the data well: *C*∼CV_force_^−0.7^ (*R*^*2*^ = 0.816). This observation demonstrates that macroscopic functional changes immediately after bLVR are linked to the underlying microscopic force redistribution. Taken together, these findings suggest that the introduction of high-force elements plays a critical role in lung volume reduction efficacy and is dependent on pre-treatment structure for LVRS but not bLVR.

### Long-Term Progressive Changes in Structure and Function

**Fig 3:**
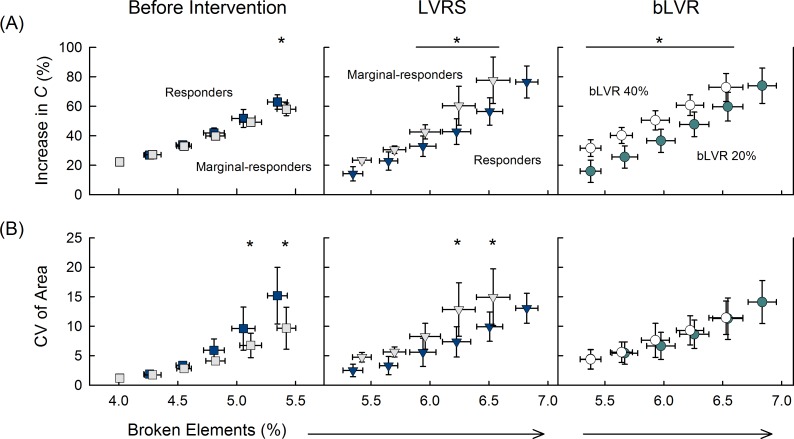
Long-term Progressive Changes in Structure and Function. Network parameters for emphysema progression before intervention (*left*) and then following LVRS (*center*) and bLVR (*right*). Mean values are shown for (A) increases in compliance, *C*; and (B) the coefficient of variation for airspace size. Disease progression was characterized by the cumulative number of broken elements and shown as a percentage of the total number of elements in the network, error bars represent standard deviation. Networks were divided before treatment (squares) and after LVRS (triangles) to illustrate differences between LVRS responders (*N* = 8, dark blue) and marginal-responders (*N* = 6, grey). All networks (*N* = 14) shown for bLVR reduction to 20% (green circles) and 40% (open circles). *Indicates statistical differences between groups.

In addition to the immediate response following lung volume reduction, we also evaluated the long-term response for both LVRS and bLVR. Disease progression was modeled as consecutive stages of increasing network deterioration characterized by the cumulative number of broken elements at each stage. Fig 3 shows the average changes in *C* and CV_area_ before and after intervention.

Prior to lung volume reduction, average values of *C* and CV_area_ increased with worsening disease severity consistent with a softening network and expanding emphysematous regions. Comparing networks classified as either responders or marginal-responders, statistically significant differences between groups were detected for *C* and CV_area_ just before intervention, suggesting that responders were slightly less elastic with more heterogeneous disease progression. Following LVRS, average values of *C* and CV_area_ initially returned to near baseline levels. Responders demonstrated smaller increases in *C* and CV_area_ at advanced disease stages indicating more sustainable functional and structural improvements compared with marginal-responders. This highlights the improved long-term treatment efficacy in networks with smaller vs. larger β, given similar stages of tissue deterioration prior to intervention. Following bLVR, no differences were detected between networks classified as either responders or marginal-responders. Instead, long-term response was related to bLVR reduction size. Networks with affected regions reduced to 20% of their original size yielded smaller increases in *C* at each stage of disease progression compared with those reduced to 40%, while average values of CV_area_ were not statistically different between groups.

### Predicted Outcomes of Survival and Quality of Life

**Fig 4:**
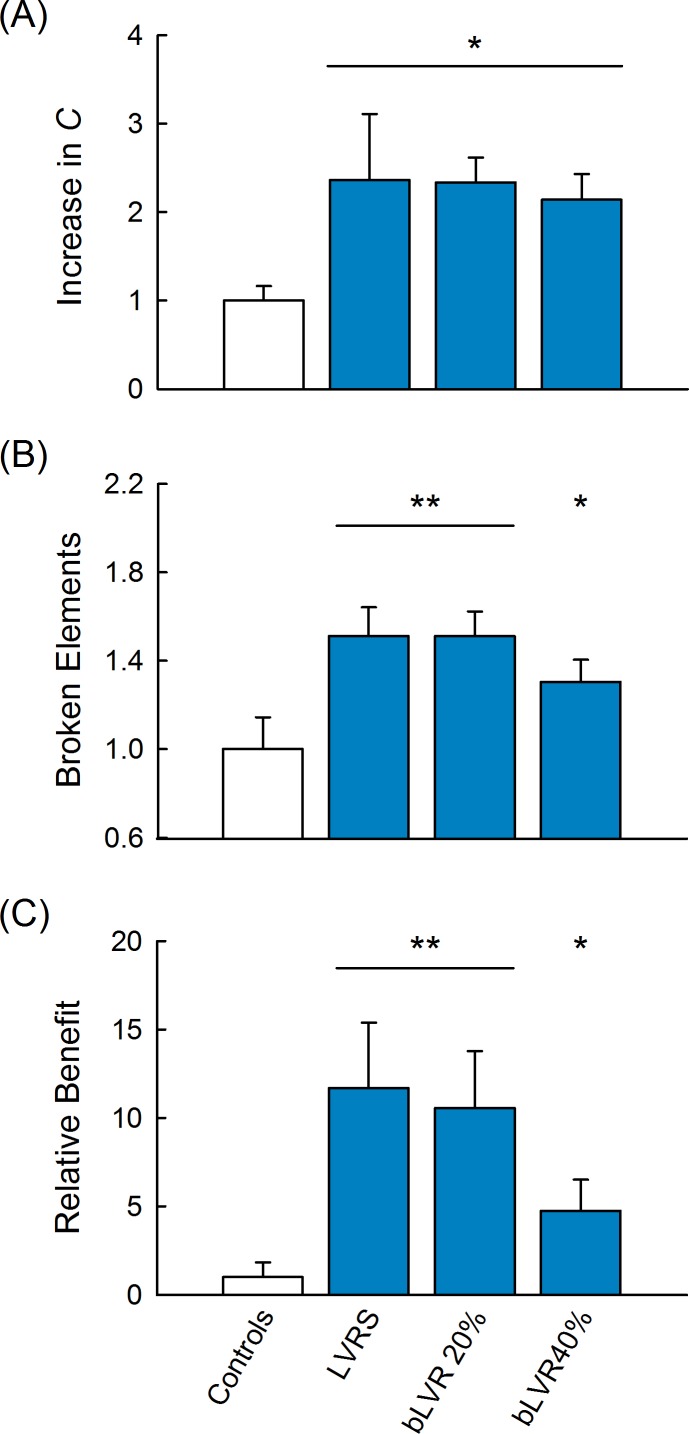
Outcome Predictions Following Lung Volume Reduction. (A) Lung volume reduction accelerated the rate of tissue failure as estimated by the increase in compliance, *C* after four steps of disease progression. (B) LVRS and bLVR reduction to 20% yielded the longest predicted survival estimated as the number of broken elements to reach a 60% increase in *C* from baseline. (C) Even greater improvements were observed for the relative benefit index, which represented a measure of quality of life. For all cases, mean values for LVRS and bLVR reduction to 20 or 40% (blue bars) were normalized by projected outcomes without treatment (open bars), error bars represent standard deviations. *Indicates values significantly larger than controls, **indicates values significantly higher than controls and bLVR reduction to 40%.

**Fig 5:**
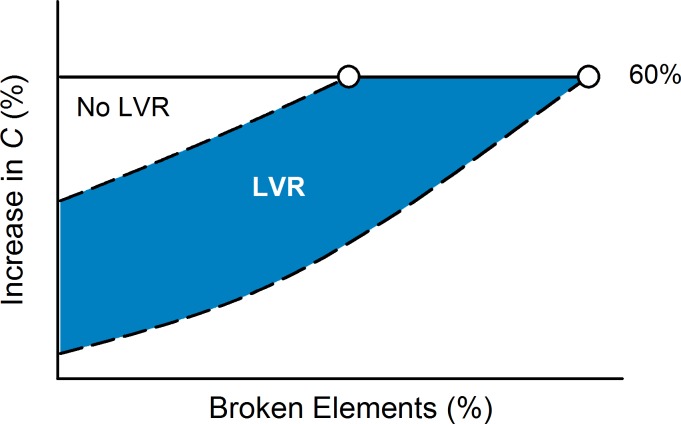
Estimation of Survival and Quality of Life. Dashed curves represent changes in compliance, *C* with and without lung volume reduction. Predicted survival was estimated as the number of broken elements to reach a 60% increase in *C* from baseline (open circles). The combined relative benefit index, which represented a clinical measure of quality of life, was defined as the area between the dashed curves and the threshold line (solid line), such that larger values corresponded to greater improvements following lung volume reduction. The area representing simulations without lung volume reduction (open region) was normalized to unity for comparison with lung volume reduction (shaded region).

Based on the long-term simulations in this computational model, we compared the predicted outcomes for LVRS and bLVR as influenced by lung compliance. We found that all treatments actually accelerated network deterioration, more than doubling the rate of increase in *C* prior to intervention (Fig 4A). Despite the accelerated rate of increase, treatments restoring *C* to near baseline levels lengthened the expected survival estimated as the number of broken elements required to reach a 60% increase from baseline. LVRS and bLVR reduction to 20% yielded increases of 1.51±0.13 and 1.51±0.11 times longer than without treatment, respectively (Fig 4B). We also calculated a combined index related to the area enclosed by the threshold and the curve defined by values of *C* (schematic shown in Fig 5). By incorporating both the rate of increase and the number of broken elements, this index termed “Relative Benefit” represented a measure for quality of life. LVRS and bLVR reduction to 20% yielded similar increases of 11.7±3.7 and 10.6±3.2 relative to without treatment, respectively, while bLVR reduction to 40% yielded a considerably smaller increase of 4.7±1.8 (Fig 4C). These model predictions indicate that bLVR can yield similar outcomes as LVRS when affected regions are appropriately reduced in size.

## Discussion

Lung volume reduction represents the primary therapeutic strategy for advanced emphysema. LVRS is a well-established surgical treatment, but is limited by strict indications and significant post-procedural complications. Several non-surgical bLVR approaches are on the rise providing less invasive alternatives with the potential for considerably lower post-procedural morbidity and mortality. While previous work has evaluated improvements in clinical function and survival advantage provided by these techniques, little is known about the corresponding micromechanical mechanisms responsible for improvement in survival and quality of life. In this study, we constructed 2D elastic networks to simulate lung volume reduction with LVRS and bLVR in a force-based model of emphysema progression. Our main findings include: (1) analysis of network structure using a simple measure of disease heterogeneity prior to lung volume reduction can predict LVRS efficacy; (2) macroscopic functional improvements following bLVR correspond to microscopic changes in force heterogeneity; and (3) lung volume reduction improves aspects of the predicted survival and quality of life influenced by contributions of lung compliance, albeit while accelerating disease progression.

Mechanical forces have long been suggested to play a role in emphysema progression [23]. Previous work [24] provided early evidence demonstrating alveolar wall rupture in elastase-treated tissue slices as a direct result of local mechanical forces. Subsequently, it was shown that increases in lung compliance paralleled changes in airspace heterogeneity associated with force-induced failure of the extracellular matrix (ECM) [25]. Inflammatory processes, concerted action of proteases, and ECM remodeling also likely contribute to emphysema progression [26–29]; however, their role has been proposed more broadly within self-propagating dynamic loops of enzymatically initiated but mechanically driven tissue destruction [19]. Previous network model simulations have demonstrated that emphysematous tissue breakdown cannot be reproduced by a purely chemical process, such that the inclusion of local forces are critical in developing observed emphysema patterns [18,19]. Alternative models based on uniform softening or random cutting have also been found to poorly characterize these progressive changes [25]. Thus, we consider the network model described here to provide a suitable description of emphysema progression with the capacity for studying the structure-function relations following lung volume reduction.

It is known that patients with predominately upper-lung emphysema have more favorable outcomes following LVRS [3,9]. In this study, greater improvements in *C* after LVRS were observed in those with less affected lower network regions (Fig 2A) as resection of the upper diseased airspaces allowed for the remaining tissue to restore function. These networks also displayed smaller increases in *C*, especially at advanced disease stages (Fig 3A), as well as considerably smaller decreases in total network stress, σ, that may reflect transpulmonary pressure in the lung (S3 Fig). This is consistent with previous experimental data demonstrating that improvements after LVRS are correlated with increased ratios between upper to lower zone emphysema (determined by computed tomography, CT), but are not well predicted by pre-surgical measurements of static lung compliance or elastic recoil [30,31]. The observed improvements in *C* may also be related to differences in force distribution between LVRS groups (Fig 2B). One possible explanation is that emphysematous areas remaining after LVRS act as “shock-absorbers” contributing to a softer overall tissue, whereas networks with relatively low structural heterogeneity better facilitate force propagation and a stiffer overall tissue. Moreover, we found that a power law distribution could be used to characterize the distribution of forces before and after intervention (S4 Fig). The tail of the distribution varied with the specific intervention and may indicate the emergence of complex network behavior [32], which would be a unique case when increased lung heterogeneity potentially contributes positively to treatment outcome. Nonetheless, these findings suggest a mechanism that may explain how functional changes evolve based on intrinsic structural differences prior to LVRS.

Motivated by the benefits observed with LVRS, however, non-surgical bronchoscopic alternatives have been the focus of recent investigations, where patient outcomes have improved as bLVR techniques have become more proficient [8,10]. A recent study using endobronchial valves [14] reported that improvements in measured FEV_1_ were correlated with effective collapse of the affected lobe, a finding confirmed to be enhanced by fissure completeness and absence of interlobar collateral ventilation [6]. This is conceptually similar to our model predictions that bLVR reduction size is inversely related to immediate and long-term improvements in *C* (Fig 2 and Fig 3). Comparing bLVR for multiple reduction sizes revealed that macroscopic functional improvements in *C* were linked to underlying microscopic changes in force heterogeneity. Although radiographic evidence indicates near complete reduction is currently not achievable [8,12,15], our findings highlight important structure-function interactions between network reorganization after bLVR and its effect on the local mechanical environment in the lung (observations which would otherwise be impossible to detect via imaging or functional studies). This unexpected relationship demonstrates that bLVR can be an effective treatment for advanced emphysema, but also suggests a mechanism by which elevated forces in close proximity to reduced areas may promote local tissue destruction.

By simulating LVRS and bLVR in parallel from the same configuration, we were able to directly contrast outcomes and disease progression after treatment. In general, lung volume reduction led to more rapid tissue failure as a result of increased mechanical forces on elastic elements. This is consistent with clinical reports of accelerated deterioration of lung function (relative to pre-surgery) observed in patients following LVRS [33,34]. Despite elevated rates of tissue failure, LVRS and bLVR are predicted to lengthen survival and improve quality of life by restoring lung function to levels closer to healthy tissue (Fig 4). Statistically comparable outcomes were observed for LVRS and bLVR reduction to 20%, suggesting that bLVR is capable of similar treatment efficacy as current surgical standards. The modality-specific reduction in bLVR efficacy highlighted by Ingenito et al. [10] might also be explained by the less proficient treatment modeled by bLVR reduction to 40%. Nonetheless, these computational findings support bLVR application across an even broader treatment population, as suggested by Deslee et al. [35]. This is of particular interest given the potential for substantially less invasive bronchoscopic techniques to extend treatment options to those who do not qualify for LVRS.

Changes in relative lung volumes are also correlated with treatment efficacy. Fessler et al. [7] have shown that decreases in residual volume (RV) relative to total lung capacity (TLC) contribute to improvements in FEV_1_ after LVRS. Our results support these findings illustrating that LVRS in heterogeneous networks and bLVR applied to affected regions represent treatments that come closest to the removal of pure RV and allow for expansion of more normal regions. Interestingly, related studies evaluating the success of bilateral lung transplant somewhat counterintuitively observed significantly better outcomes in cases with donor lungs larger than the recipient thorax (as estimated by the donor-recipient predicted TLC ratio) [36,37]. It was proposed, however, that a decrease in lung compliance post-transplant was likely a contributing factor for survival and performance, which would agree with the benefits of lung volume reduction modeled here. The importance of these anatomic considerations also suggests the potential for coupling this computational approach with CT imaging in the future. The non-invasiveness of such an analysis would be uniquely suited to infer patient outcomes prior to treatment and aid clinical decision-making.

There are several limitations of the network model that must be considered when associating computational findings with clinical outcomes. (1) Our model considers the lung tissue to be a collection of interconnected acinar compartments; however, recent work has highlighted the involvement of the small airways in COPD development. Narrowing and loss of terminal bronchioles are believed to increase small airway resistance in COPD patients [38], and may even precede emphysema development [39]. Hiorns et al. [40] have further demonstrated the dynamic and spatially heterogeneous nature of these airway-parenchyma tethering interactions in precision cut lung slices. Although interactions at this scale are not included here, the stiffer airways would likely be associated with local parenchymal destruction. The hexagonal network units might alternatively reflect the mechanics of secondary pulmonary lobules, approximating the coalescence of destroyed lobules and enlarged lesions characteristic of emphysema, as described by Hogg et al. [41]. (2) The number of broken elements may not have a direct temporal correlation with progression *in vivo* even though tissue destruction is clearly associated with more developed disease severity. Moreover, emphysema progression is modeled by breaking a specified number of elements at each stage of disease. An alternative approach would be to define a global threshold, as investigated for ventilator-induced lung damage [42], above which elements are considered to fail. Both approaches yield similar disease patterns, but could influence the apparent rate of tissue failure differently. Nonetheless, observed inter- and intra-subject variability *in vivo*, along with only few data from follow-up studies, validate the general interpretations of our results presented here. (3) Ventilatory dependencies associated with incomplete lung fissures are not captured by our network models; however, the bLVR simulations presented here are markedly similar to the mechanical action of nitinol coils and biomaterial-based lung sealants believed to function independently of collateral ventilation pathways [12,15]. (4) Chest wall mechanics, irregular lung boundaries, nonlinear dynamics, 3D interactions, enzyme kinetics, and ECM remodeling were also not included in this study, but could improve the interpretation of factors contributing to disease progression. Future work expanding bLVR in a true multiscale model of emphysema in 3D [43] that incorporates airway-parenchymal interactions, inflammation, and enzyme kinetics may provide additional insights and enhance the potential for clinical application. Despite these limitations our network model has been shown to generate disease patterns with strong correlation to those observed using CT imaging [18] by including contributions of mechanical forces that likely drive emphysema progression [19].

While these computational simulations represent a simplified view of emphysema progression, this model provides new perspective into the structure-function relations underlying the progressive nature of emphysema before and after lung volume reduction. Immediate and long-term responses to these interventions appear to be intimately linked to changes in microscopic force heterogeneity within the lung, which could explain known structural limitations for surgical approaches and emphasize pertinent implications in disease progression for bronchoscopic approaches. Furthermore, our findings suggest that effective bronchoscopic reduction of affected lung tissue can achieve similar if not better functional improvements, survival advantages, and quality of life benefits as currently established surgical techniques. These insights have the potential to inform more rationalized design of lung volume reduction techniques and patient-specific treatment strategies.

## Methods

### Experimental Design

We constructed *N* = 14 networks to model the elastic behavior of the lung parenchyma with different initial conditions simulating inter-subject variability. Networks were progressively degraded by eliminating elements carrying the highest forces and then finding the network configuration with minimal elastic energy for five sequential iterations. LVRS and bLVR were then applied to the same network configuration and the treated networks were subsequently degraded as before. Structural and functional parameters were tracked for each network configuration to characterize changes at each stage of disease progression. Finally, predicted survival and quality of life outcomes were compared for both treatments.

### Elastic Network Model

The 2D network model used in this study has been described previously [18–22]. Briefly, elastic elements inter-connected via pin joints were allowed to rotate freely while nodes bordering the perimeter of the network were kept fixed to ensure the network was initially pre-stressed and hexagonal units, representing individual acini, were not collapsed. For each configuration, the total elastic energy, *E*_*tot*_ was calculated as the sum of the energies for individual elastic elements, *E*_*i*_:

$$E_{tot}=\sum_{i}E_{i}=\frac{1}{2}k_{i}{\Delta l}_{i}^{2}$$

where *k*_*i*_ are the linear spring constants and *Δl*_*i*_ are the individual element displacements from their resting length. Each network consisted of 6,987 elastic elements and 2,310 hexagonal cells (85x56 nodes). Uniform distributions (Mean ± SD) of spring constants (1.0 ± 0.4) and resting lengths (0.5 ± 0.1) were assigned to the elastic elements to introduce a degree of initial heterogeneity.

The minimum energy corresponding to the equilibrium configuration of the network was obtained using the equation above with a variant of the simulated annealing technique [44,45]. Here, the position of each node was displaced by a small amount proportional to and in the direction of the local resulting force on the node. If the change in total energy compared to the previous position was negative (*ΔE* < 0) the new configuration representing a lower energy state of the system was accepted. Alternatively, for *ΔE* ≥ 0, the new configuration could be accepted with probability *P* = *exp*(−Δ*E*/*T*) where *T* was a control parameter that was sequentially reduced until a pre-defined convergence criterion was reached.

Gravity dependence in the network was simulated by applying additional downward forces at each node with magnitude proportional to the number of dependent nodes below. This relatively weak influence represented the net effect of gravity over long time-scales proposed to enhance tissue destruction in the upper lung [23]. In the absence of this term, emphysema would be expected to progress with equal probability in any region of the network.

### Force-Based Emphysema Progression and Lung Volume Reduction

Emphysema was initiated in the network model by randomly breaking ~4% of all the elastic elements. Tissue failure was then simulated using a force-based destruction approach. Elastic elements were sorted by their corresponding force, and the top 0.7% of elements were broken with probability *P* = 0.40. Individual elements were not considered to experience fatigue behavior. The modified network was solved to yield a new configuration and distribution of forces, which corresponded to a later disease stage with different elastic elements at risk for failure. This discretized approach generated a disease progression driven by the spatial distribution of forces while the probabilistic elimination of elements introduced a degree of stochasticity to each network, limiting the deterministic nature of each simulation. These steps were repeated for a total of five iterations simulating progressively more developed disease severity.

LVRS and bLVR were applied in parallel to reduce affected emphysematous regions. To simulate lung resection in LVRS, the upper 30% of the network was removed and affected regions intersected by the threshold were stretched to form a continuous, fixed horizontal upper border. To simulate reduction of enlarged airspaces in bLVR, nodes encompassed by a selected perimeter, corresponding to the region to be reduced, were moved toward their geometric center of mass. Regions including a fixed border were asymmetrically reduced in size parallel to the axis of the border. For each network, affected regions were selected and then reduced to 1, 20, or 40% of their original size.

### Estimation of Functional and Structural Network Parameters

Mechanical stress σ was calculated for each network configuration by numerically differentiating the total energy, *E*_*tot*_ of the system at the equilibrium configuration and after stretching the network by a small bi-axial strain, ε = ±0.01. Here, the equilibrium configuration was assumed to correspond to FRC, representing a static measurement of lung function. Networks were then stretched with a sinusoid of amplitude ε = ±0.04 around the equilibrium configuration, such that the 2D bulk modulus was defined as the slope of the corresponding stress-strain curve. The compliance *C* was calculated as the inverse of the estimated network bulk modulus at each stage of disease progression. To facilitate comparisons with baseline, σ and *C* are reported as percent changes from the initial network configuration prior to emphysema destruction.

Network structure was quantified by considering the sizes of individual airspace units. Each network configuration was converted to a binary image and the number of pixels enclosed by connected spring elements represented the individual airspace area. Overall structural heterogeneity was then assessed as the coefficient of variation of airspace sizes, CV_area_. For network configurations directly before intervention, we also considered the coefficient of variation for airspaces below the line of LVRS resection. This predictive index, referred to as β, subsequently characterized disease heterogeneity in the network not resected by LVRS. Note that β was calculated as a single predictive index before treatment, whereas CV_area_ was calculated for each stage of disease progression to track changes in overall network structure.

### LVRS and bLVR Outcome Predictions

The rate of tissue failure was estimated before and after intervention as the increase in *C* over four stages of disease progression. The number of broken springs required to reach a 60% increase in *C* was calculated for each network as an estimate of survival. However, since network deterioration prior to treatment was typically less than this threshold a second order polynomial was fitted to values of *C* to estimate survival in the absence of any lung volume reduction. The relative benefit of treatment was then calculated as shown in the schematic (Fig 5). The area between the survival threshold and the compliance curve represents a composite index for quality of life, incorporating both the rate and sub-threshold duration of disease progression. Larger values of this area correspond to lower values of *C* over a longer period of time and hence represent better quality of life. To compare the benefits provided by lung volume reduction, data are reported as normalized by the estimated values in the absence of any treatment.

### Simulations

Network simulations were completed using custom-developed software, which has been utilized previously to generate and analyze networks in conjunction with other experimental studies [18–22]. Network manipulations involving LVRS and bLVR were implemented cooperatively with this program using original scripts developed in MATLAB (MATLAB r2013a, MathWorks, Natick, MA).

### Data Analysis

Two-way repeated measure analysis of variance (ANOVA) was used to compare network values of *C*, CV_area_, and σ between treatment groups at each stage of disease progression, as well as the skewness of force distributions for each treatment group. One-way ANOVA was used to compare estimates of disease progression rate, survival, and relative benefit. Post-hoc Holm-Sidak and Tukey tests were used to determine differences between groups. The average change in *C* after LVRS for responder and marginal-responders were compared using a t-test. For all comparisons, *p*<0.05 was considered significant. Statistical analyses were performed using SigmaPlot (SigmaPlot v12.3, Systat Software, Inc., San Jose, CA) and MATLAB.

## Supporting Information

S1 Fig Lung Volume Reduction in a Marginal-responder Network. (A) Representative simulation of emphysema progression before intervention, and (B) comparison of lung volume reduction techniques in a marginal-responder network. See Fig 1 for additional details on sequence of individual panels. (C) Changes in compliance, *C* for the representative network shown.
(TIF)

S2 Fig Structural Heterogeneity in LVRS Networks. (A) Representative responder (*left*) and marginal-responder (*right*) configurations prior to LVRS. Tissue heterogeneity below the line of resection was characterized by the predictive index β (see definition in the main text), while CV_area_ represented heterogeneity throughout the entire network. Blue shading indicates larger, affected regions not removed by LVRS (shaded upper region). (B) Prior to LVRS, responders were characterized by greater overall heterogeneity (*i*.*e*., larger CV_area_; *p* = 0.036) limited to the upper network region (*i*.*e*., smaller β; *p*<0.001) as compared with marginal-responders.
(TIF)

S3 Fig Long-term Progressive Changes in Network Stress. Mean values for drop in σ during emphysema progression before intervention (*left*) and then following LVRS (*center*) and bLVR (*right*). Changes in σ may reflect variations in transpulmonary pressure for the system at FRC. Disease progression was characterized by the cumulative number of broken elements and shown as a percentage of the total number of elements in the network, error bars represent standard deviation. Networks were divided before treatment (squares) and after LVRS (triangles) to illustrate differences between LVRS responders (*N* = 8, dark blue) and marginal-responders (*N* = 6, grey). All networks (*N* = 14) shown for bLVR reduction to 20% (green circles) and 40% (open circles). *Indicates statistical differences between groups.
(TIF)

S4 Fig Power Law Analysis of Responses to Lung Volume Reduction. For each treatment group, we calculated the histogram of forces across all networks using log-spaced binning, scaled the counts by the corresponding bin width, and then normalized the area under the curve to unity to obtain the probability density function. A simple power law function, Probability ~ Force^α^, was fitted to the linear portion of the data corresponding to the tails of the distributions plotted on a log-log graph. (A) Probability density functions of force distribution directly before and after lung volume reduction. (B) Values of exponent α, root mean squared error (RMSE), and *R*^*2*^ for the simple power law functions fitted to the data. Note that the magnitude of α decreases for treatment groups with larger immediate drops in *C*, suggesting that more heavily skewed force distributions may contribute to functional improvements after lung volume reduction. For marginal-responders, the inability to introduce high force element after LVRS may explain the observation of a softer overall tissue and smaller changes in *C*. Interestingly, this would indicate a rare occurrence when heterogeneity in the lung is beneficial for amelioration of disease condition.
(TIF)
