# Supplementary figures and images for: Predicting Structure-Function Relations and Survival following Surgical and Bronchoscopic Lung Volume Reduction Treatment of Emphysema
Source: PLoS Comput Biol. 2017 Feb 9;13(2):e1005282. doi: 10.1371/journal.pcbi.1005282 (PMC5300131; doi:10.1371/journal.pcbi.1005282)

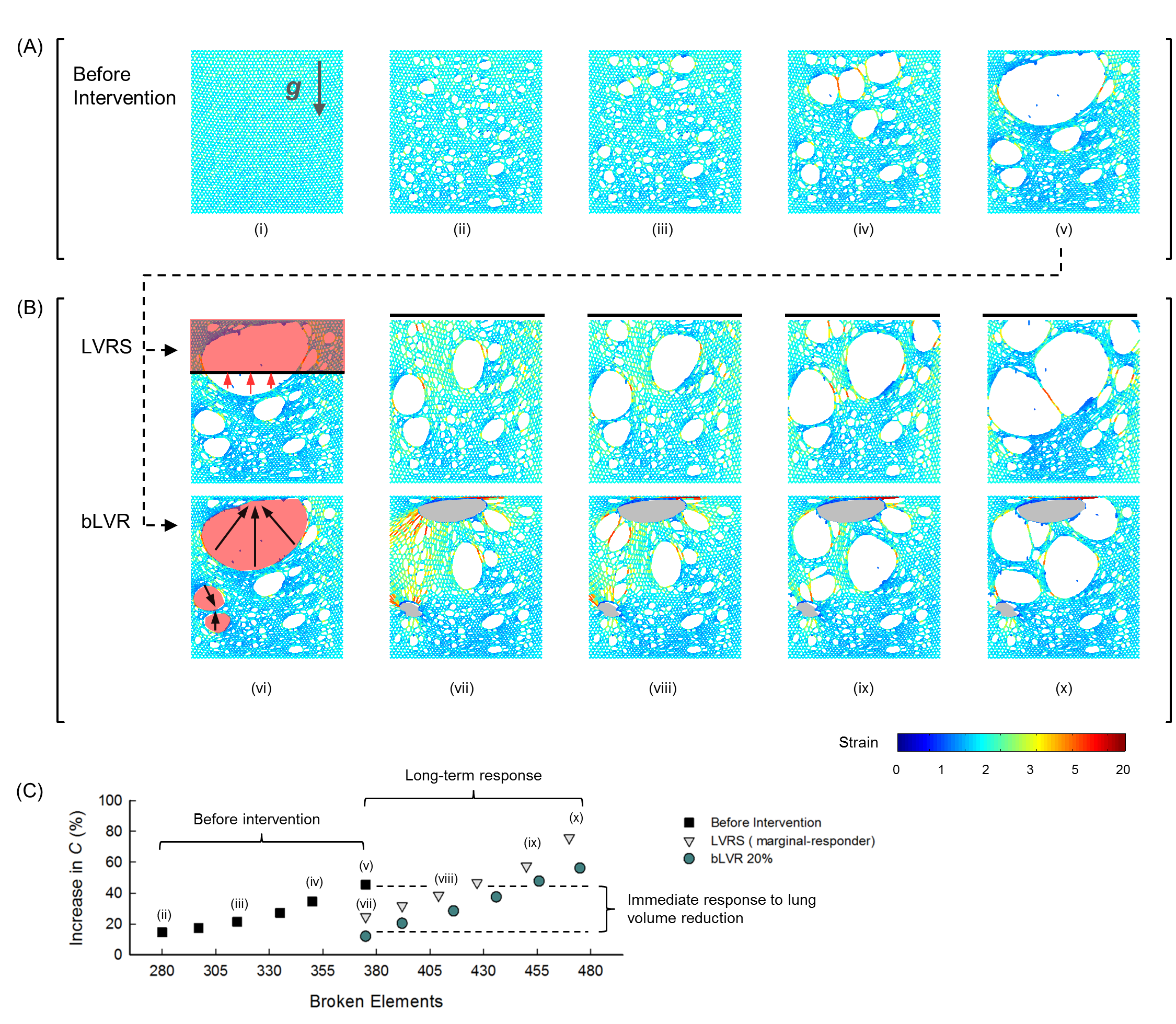

Supplement: S1 Fig — (A) Representative simulation of emphysema progression before intervention, and (B) comparison of lung volume reduction techniques in a marginal-responder network. See Fig 1 for additional details on sequence of individual panels. (C) Changes in compliance, C for the representative network shown. (TIF) [file pcbi.1005282.s001.TIF]

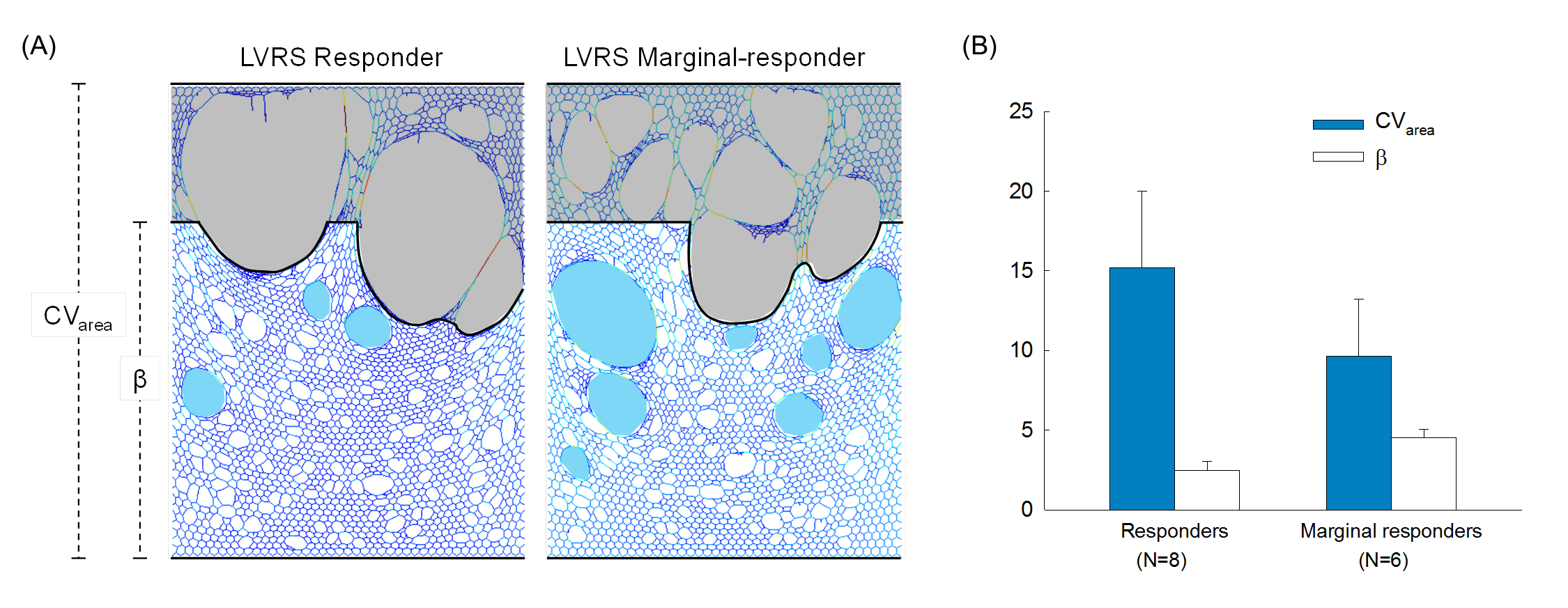

Supplement: S2 Fig — (A) Representative responder (left) and marginal-responder (right) configurations prior to LVRS. Tissue heterogeneity below the line of resection was characterized by the predictive index β (see definition in the main text), while CVarea represented heterogeneity throughout the entire network. Blue shading indicates larger, affected regions not removed by LVRS (shaded upper region). (B) Prior to LVRS, responders were characterized by greater overall heterogeneity (i.e., larger CVarea; p = 0.036) limited to the upper network region (i.e., smaller β; p<0.001) as compared with marginal-responders. (TIF) [file pcbi.1005282.s002.TIF]

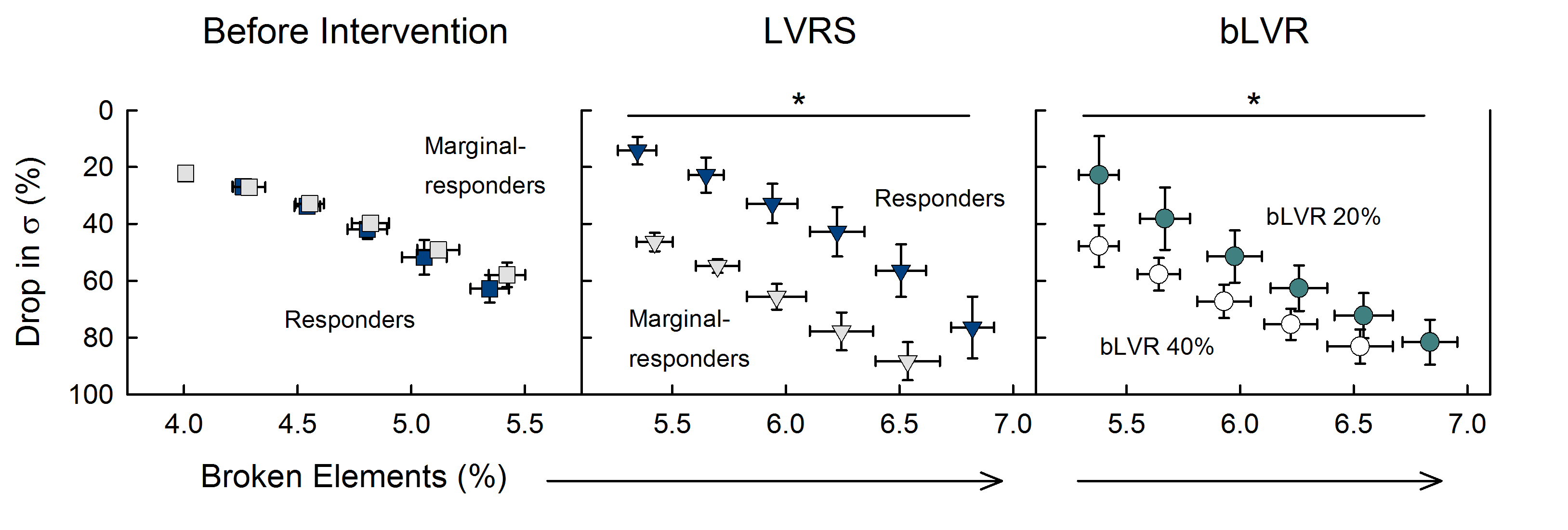

Supplement: S3 Fig — Mean values for drop in σ during emphysema progression before intervention (left) and then following LVRS (center) and bLVR (right). Changes in σ may reflect variations in transpulmonary pressure for the system at FRC. Disease progression was characterized by the cumulative number of broken elements and shown as a percentage of the total number of elements in the network, error bars represent standard deviation. Networks were divided before treatment (squares) and after LVRS (triangles) to illustrate differences between LVRS responders (N = 8, dark blue) and marginal-responders (N = 6, grey). All networks (N = 14) shown for bLVR reduction to 20% (green circles) and 40% (open circles). *Indicates statistical differences between groups. (TIF) [file pcbi.1005282.s003.TIF]

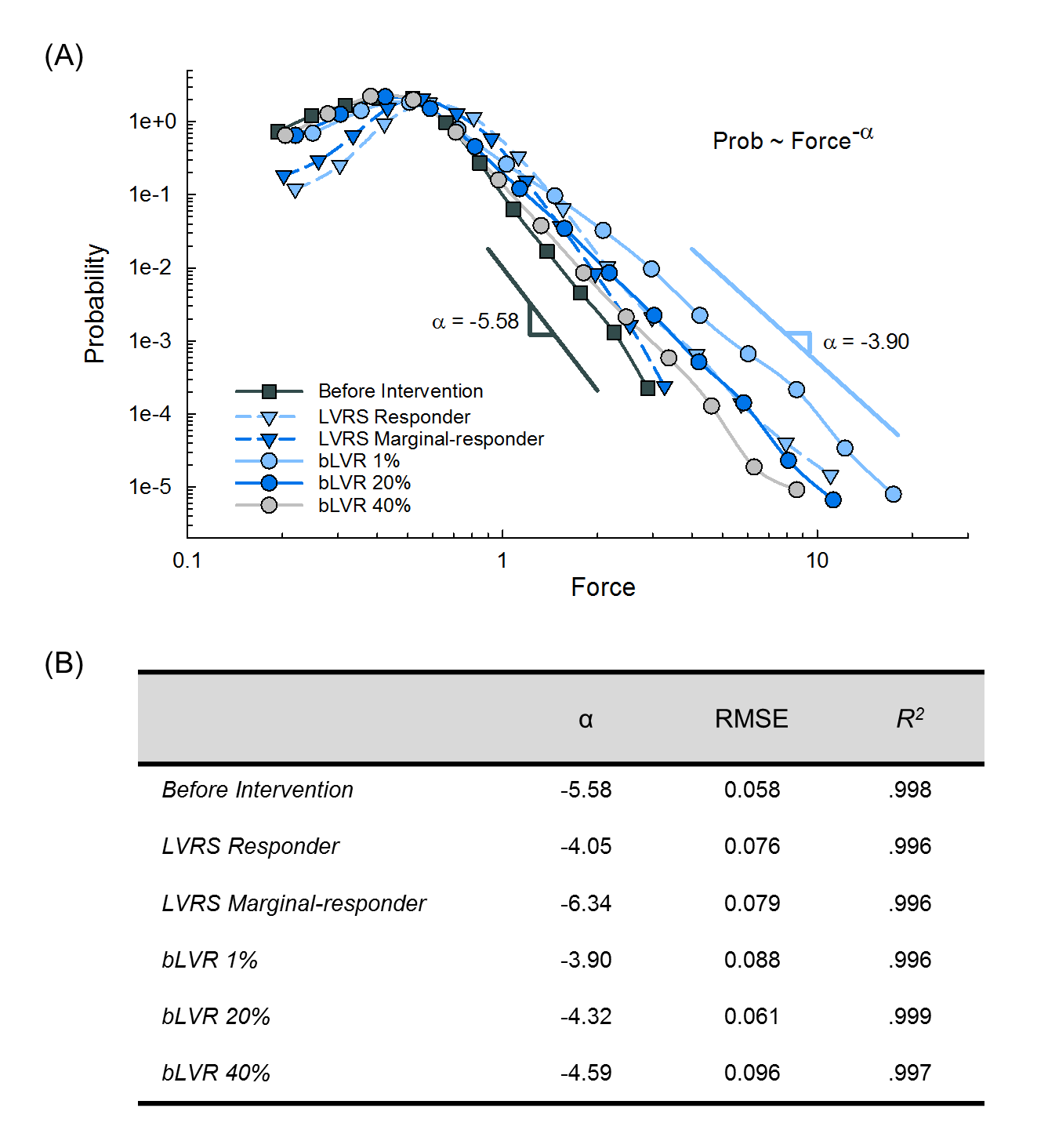

Supplement: S4 Fig — For each treatment group, we calculated the histogram of forces across all networks using log-spaced binning, scaled the counts by the corresponding bin width, and then normalized the area under the curve to unity to obtain the probability density function. A simple power law function, Probability ~ Forceα, was fitted to the linear portion of the data corresponding to the tails of the distributions plotted on a log-log graph. (A) Probability density functions of force distribution directly before and after lung volume reduction. (B) Values of exponent α, root mean squared error (RMSE), and R2 for the simple power law functions fitted to the data. Note that the magnitude of α decreases for treatment groups with larger immediate drops in C, suggesting that more heavily skewed force distributions may contribute to functional improvements after lung volume reduction. For marginal-responders, the inability to introduce high force element after LVRS may explain the observation of a softer overall tissue and smaller changes in C. Interestingly, this would indicate a rare occurrence when heterogeneity in the lung is beneficial for amelioration of disease condition. (TIF) [file pcbi.1005282.s004.TIF]
